# Virus Excretion from Foot-And-Mouth Disease Virus Carrier Cattle and Their Potential Role in Causing New Outbreaks

**DOI:** 10.1371/journal.pone.0128815

**Published:** 2015-06-25

**Authors:** Aravindh Babu R. Parthiban, Mana Mahapatra, Simon Gubbins, Satya Parida

**Affiliations:** The Pirbright Institute, Ash Road, Pirbright, Surrey GU24 0NF, United Kingdom; Institut National de la Santé et de la Recherche Médicale (INSERM), FRANCE

## Abstract

The role of foot-and-mouth disease virus (FMDV) carrier cattle in causing new outbreaks is still a matter of debate and it is important to find out these carrier animals by post-outbreak serosurveillance to declare freedom from FMDV infection. In this study we explore the differences in viral shedding between carrier and non-carrier animals, quantify the transmission rate of FMDV infection from carriers to susceptible animals and identify potential viral determinants of viral persistence. We collected nasal and saliva samples from 32 vaccinated and 7 unvaccinated FMDV carrier cattle and 48 vaccinated and 13 unvaccinated non-carrier cattle (total n=100) during the acute phase of infection (up to 28 days post-challenge) and then from limited number of animals up to a maximum 168 days post-challenge. We demonstrate that unvaccinated cattle excrete significantly higher levels of virus for longer periods compared with vaccinated cattle and this is independent of whether or not they subsequently become carriers. By introducing naïve cattle in to the FMDV carrier population we show the risk of new outbreaks is clearly very low in controlled conditions, although there could still be a potential threat of these carrier animals causing new outbreaks in the field situation. Finally, we compared the complete genome sequences of viruses from carrier cattle with the challenge virus and found no evidence for viral determinants of the carrier state.

## Introduction

Foot-and-mouth disease (FMD) is a highly contagious, acute viral disease of cloven-hoofed animals, characterized by fever, loss of appetite, depression, lameness and the appearance of vesicles on the feet and in, or around, the mouth. Infected cattle generally clear the systemic infection within 8–15 days [1]. However, FMD virus (FMDV) can persist in the oropharynx for years following the resolution of acute infection [2]. Animals from which live-virus can be recovered 28 days post infection are defined as persistently-infected [3] and up to 50% of FMD-recovered ruminants become persistently infected, irrespective of their vaccination status [4]. By contrast, pigs usually clear FMDV within 3 weeks following infection and do not become carriers [5, 6, 7].

After clearing from blood and all affected sites, the virus can be found in the nasopharynx of persistently infected ruminants and is associated with the basal layers of the epithelium [8, 9]. The mechanisms of persistence of FMDV in host species have not been described, although immune responses, both cellular and humoral, and cytokine responses have been suggested to play a critical role [10, 11]. There is experimental evidence that increasing vaccine dose may reduce virus excretion and may also reduce the frequency of carriers in vaccinated cattle [12, 13]. This is confirmed by the observation that the probability of an animal becoming a carrier is higher in groups receiving a lower vaccine dose [12, 13].

The role of carriers in the occurrence of new FMD outbreaks is a matter of debate [14]. There is circumstantial evidence that carriers may have been the occasional cause of outbreaks [2, 15, 16, 17, 18, 19] but other routes of introductions could not be excluded in these observational studies [20]. In this study we investigated the carrier state in FMDV-infected cattle in vaccine-challenge experiments. In particular, we explored three questions. First, what are the differences in viral shedding between carrier and non-carrier animals during the acute phase of infection (i.e. could we determine which animals are likely to become carriers)? Second, what is the probability of transmission from carriers to naive cattle under controlled conditions (i.e. how much of a risk do carriers pose of initiating outbreaks)? Third, what, if any, are the differences between the virus used to challenge the animals and those recovered from carrier animals at different times post infection (i.e. are there viral determinants of the carrier state)?

## Materials and Methods

### Vaccine-challenge and transmission experiments

Four FMDV vaccine-challenge experiments were previously conducted in BSL3 containment isolation units at The Pirbright Institute to study FMDV infection in vaccinated animals [12, 13, 21, 22, 23]. All the animal experiments were conducted in accordance with UK Home Office (HO) Rules and approved by The Pirbright Institute Ethics Committee. Procedures and end-points were defined in HO Project Licenses (PPL 70/5900) and were conducted by qualified persons as specified in their Personal HO Licenses. In each experiment, 20 cattle were vaccinated with oil formulated FMDV type O1 Manisa emergency vaccine obtained from the UK-FMDV antigen reserve. All vaccinated and unvaccinated cattle (n = 5 in each experiment) in all the four experiments were directly exposed to a heterologous O UKG 34/2001 FMD virus either at 21 days [12, 21] or at 10 days [23] post vaccination by mixing the vaccinated and unvaccinated control cattle for five days with the donor infected cattle. Donor cattle had been challenged on the previous day by intradermolingual inoculation of 10^5^ TCID_50_ O UKG 34/2001 FMD virus. The donor and non-vaccinated control cattle were removed at the end of the five-day contact period and the non-vaccinated control cattle were maintained separately from the vaccinated cattle.

All the vaccinated and non-vaccinated challenged cattle of the first experiment (1x antigen Payload and 21 days vaccination) were kept for 28 days post challenge (dpc) and then 15 animals (12 vaccinated and 3 unvaccinated) were selected for long-term study over a further 140 days [13, 22]. Similarly, in the second experiment(10x antigen Payload and 21 days vaccination), all vaccinated and non-vaccinated cattle were kept for 42 dpc and then 14 animals (10 vaccinated and 4 unvaccinated) were selected and maintained for a further 63 days [13]. All the animals in the remaining two experiments were challenged on 10 days post-vaccination and maintained for 77 days and 35 days post challenge, at which point the experiments were terminated (Table 1). Selection of animals for long-term study was based on the positive results of virus isolation and RT-PCR of oro-pharyngeal (probang) fluids. The total number of vaccinated animals was 80 and total number of unvaccinated animals was 20. In total, there were 32 vaccinated carriers and 7 unvaccinated carriers (Table 1) [12, 13, 21, 22, 23].

**Table 1:** Details of vaccine challenge experiments and their clinical outcome.

Animal Expt.	Vaccine dose	Post-vac challenge day	Clinically infected/ Total vaccinated	Vaccinated carrier cattle detected by VI+RT-PCR	Clinically infected/ Total unvaccinated	Unvaccinated carrier cattle detected by VI+RT-PCR	Termination of experiment (Days post-challenge)
1	O1Manisa1X	21	0/20	9	5/5	0	168
2	O1Manisa 10X	21	0/20	3	5/5	3	105
3	O1Manisa 1X	10	5/20	9	5/5	1	77
4	O1Manisa 10X	10	6/20	11	5/5	3	35

From the first experiment 9 persistently infected animals were kept in two separate rooms and at 93 dpc a new FMD naïve, age-matched steer was added to each of these pens to act as a sentinel for continuing virus transmission. Another two naïve animals were introduced at 42 dpc with the 3 carriers selected from the second experiment to see if they would become infected following close contact with the persistently infected cattle. In the first experiment the animals were kept up to 168 days post challenge and in the second experiments the animals were kept up to 105 days post-challenge.

### Measurement of viral shedding by Real-Time RT-PCR

All of the 100 cattle (vaccinated and unvaccinated) were observed daily and in addition to a range of samples including blood and oro-pharyngeal fluid (probang fluid) collected at least on a weekly basis as described in the earlier publications. Sterile cotton bud swabs were used to collect nasal fluid and saliva in 200μl Roche lysis buffer more frequently (0, 2, 4, 7, 12, 16, 21 and 28 days post challenge) in the early phase of experiments and then once per week to measure the excretion of virus. Swabs were stored at -70°C, prior to RNA extraction and PCR.

Total nucleic acid was extracted from liquid samples with MagNA pure LC total nucleic acid isolation kits (Roche) using an automated nucleic acid robotic work-station (Roche) [24]. Briefly, RNA was extracted from 200μl of the original samples and a final volume of 18μl RNA was recovered at the end of the process. This material was used for real-time RT-PCR. Viral RNA in each sample was reverse transcribed [25] using random hexamers and quantified by real-time RT-PCR using primers and a probe from the internal ribosomal entry site (IRES) of FMDV OUKG 34/01 [24]. A Stratagene MX4000 PCR machine was used.

### Statistical analysis of viral shedding

Data on viral shedding (RNA copy number) in nasal fluid and in saliva over time were analysed by determining for each animal: (i) maximum copy number (i.e. peak shedding); (ii) area under the curve (AUC), computed using the trapezium rule (i.e. total shedding); and (iii) duration of shedding (defined as the interval between the midpoint of the first observation with a Ct value and the preceding negative observation and the midpoint of last observation with a Ct value and the subsequent negative observation).

The first two measures (peak and total shedding) were compared for vaccinated and unvaccinated cattle, carriers and non-carriers and, for vaccinated cattle only, carriers and non-carriers using Wilcoxon rank-sum tests. (Non-parametric tests were used because preliminary analysis indicated non-normality of the data.) Duration of shedding was analysed using a Cox proportional hazard model with duration as the dependent variable and vaccination status and carrier status as independent factors. In addition, for animals which became carriers, we estimated the proportion of animals shedding virus on days 21 and 28 post challenge, with the proportions compared using Fisher exact tests.

Finally, the force of infection for carriers was estimated using the results of the challenge study. Assuming a constant force of infection for the duration of the study the probability of the naïve sentinels not becoming infected is given by,

$$\text{Pr}(\text{no transmission})=e^{-\lambda T},$$

where λ is the force of infection and *T* is the duration of contact between the naïve sentinel and the carrier. The log likelihood for the data is given by,

$$l(\lambda )=-\lambda \sum_{i}T_{i}$$

where the summation is over all carriers in the experiment.

### Genome amplification and sequencing

The complete genomes of the challenge virus and RNA extracted from probang fluids from 3 carrier animals at 6 different dates post-challenge were sequenced and used in this study (animal UV9 at 49, 77, 84 and 98 dpc; animal UV13 at 49 dpc; and animal UV17 at 91 dpc). In addition, the capsid region was sequenced for a further 8 viruses in this study. One virus was sequenced from the probang fluid of a carrier animal in the present study (animal UV19 at 91 dpc), while 7 viruses were isolated from acutely-infected animals. The GenBank reference numbers for the sequences submitted in this study are provided in the S1 Table. In addition, capsid sequences for 16 viruses were obtained from GenBank submission, of which 10 were from acutely-infected animals during outbreaks between 1962 and 2010 and 6 were from carrier animals. These were used to explore whether an amino acid substitution at position 79 of the VP2 protein (tyrosine (Y) to histidine (H)) was associated with carrier viruses, as had been suggested in a previous study.

RNA was extracted from the inoculum used for challenge and from the probang fluids collected in lysis buffer from persistently infected animals using the QiaAMP viral RNA mini kit as per manufacturer’s instructions. The complete FMDV genome was amplified as five segments from the extracted RNA by Reverse Transcription—Polymerase Chain Reaction (RT-PCR) using the primers listed in S2 Table. The reverse transcription reaction was carried out at 50°C for 30 minutes, followed by the PCR amplification which constitutes an initial denaturation at 96°C for 2 minutes, 30 cycles of 96°C for 15 seconds, 65°C for 1 minute and 72°C for 2 minutes and a final extension of 72°C for 10 minutes. The amplified PCR products were purified using QIA quick PCR purification kit.

The forward and reverse sequencing reactions (primers listed in S3 Table) were performed in a 96 well ABI Sequencing plate using Big Dye Terminator v3.1 Cycle Sequencing Kit (ABI, Warrington, UK). The plate was run on a programme of 30 cycles of 96°C for 20 seconds, 50°C for 20 seconds, and 60°C for 4 minutes. Following thermal-cycling, the reactions were cleaned up by ethanol precipitation before resuspending in Hi-Di formamide and running on an ABI3730 DNA Analyzer (Applied Biosystems, CA, USA). The raw sequence data were assembled into contigs using SeqMan (Lasergene7.1, DNAStar Inc., WI, USA) and analysed using BioEdit 7.0.5.3 [26].

### Sequence analysis

Analyses of selection pressure across the coding region of the FMDV genome was performed by obtaining mean ratios of non-synonymous (dN) to synonymous (dS) substitutions. The proportion of synonymous substitutions per potential synonymous site and the proportion of non-synonymous substitutions per potential non-synonymous site were calculated by the method of Nei and Gojobori implemented within JCoDA software [27]. The genealogical relationship between the complete genome sequences including the untranslated regions was computed using statistical parsimony as implemented in the software package TCS v1.21 [28]. The full genome sequences of the viruses were subjected to jModelTest 0.1.1 [29] to determine the most suitable nucleotide substitution model and Bayesian analysis was performed using the BEAST software package v1.7.1 [30]. For each data-set, the maximum clade credibility (MCC) phylogenetic tree was inferred using the Bayesian Markov Chain Monte Carlo (MCMC) method. By incorporating the date of sample collection, the age of each virus was estimated. In BEAUti v1.7.1, the analysis utilised the TN93+I substitution model to describe rate heterogeneity among sites. In order to accommodate variation in substitution rate among branches, both uncorrelated lognormal relaxed clock and the random local clock models were chosen for this analysis [31]. Two chains of 10^7^ iterations were run and the output viewed with TRACER 1.5 [30]. Trees from multiple runs were combined using the LogCombiner v1.7.1 program and evolutionary trees were generated in the FigTree program v1.4.2.

## Results

### Viral shedding from carrier and non-carrier animals

**Fig 1:**
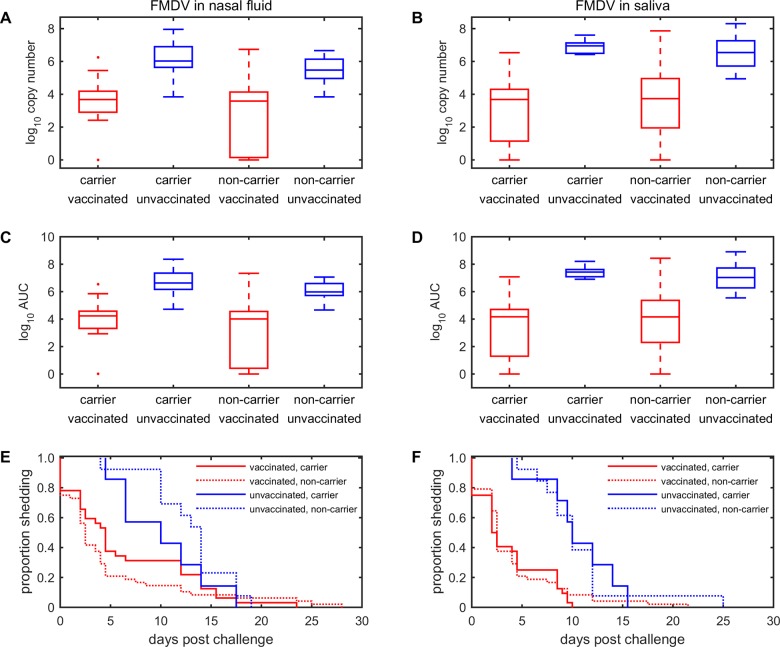
Excretion of foot-and-mouth disease virus by infected cattle and the relationship with carrier status and vaccination. (*A*,*B*) Peak shedding (log_10_ copy number). (*C*,*D*) Total shedding (log_10_ area under the curve). (*E*,F) Duration of shedding (proportion of cattle shedding over time). Viral RNA was isolated from (*A*,*C*,*E*) nasal fluid or (B,*D*,*F*) saliva by qPCR. Box-and-whisker plots show the median (line), interquartile range (box), 1.5 times the interquartile range (whiskers) and any outliers (crosses). Boxes or lines which are the same colour do not differ significantly (P>0.05) from one another, while boxes or lines which differ in colour are significantly different (P<0.001).

The median peak level of virus shedding in saliva was 10^6.9^ copies/ml in unvaccinated carriers, 10^3.7^ copies/ml in vaccinated carriers, 10^6.5^ copies/ ml in unvaccinated non-carriers and 10^3.7^ copies/ml in vaccinated non-carriers (Fig 1A). The corresponding levels in nasal fluid were 10^6.0^, 10^3.7^, 10^5.5^ and 10^3.6^ copies/ml, respectively (Fig 1B). The peak period of shedding occurred between 2 and 7 days post infection, irrespective of vaccination or carrier status (S4 and S5 Tables). The median total quantity of virus shed (i.e. area under the curve) was 10^7.4^ copies/ml in unvaccinated carriers, 10^4.2^ copies/ml in vaccinated carriers, 10^7.0^ copies/ml in unvaccinated non-carriers and 10^4.2^ copies/ml in vaccinated non-carriers (Fig 1C). The corresponding quantities in nasal fluid were 10^6.6^, 10^4.2^, 10^6.0^ and 10^4.0^ copies/ml, respectively (Fig 1D). Finally, the median duration of shedding in saliva was 10.0 days in unvaccinated carriers, 2.3 days in vaccinated carriers, 10.0 days in unvaccinated non-carriers and 2.5 days in vaccinated non-carriers (Fig 1E). The corresponding durations in nasal fluid were 10.0, 4.5, 14.0 and 2.5 days, respectively (Fig 1F).

For both nasal fluid and saliva, unvaccinated cattle excreted significantly (*P*<0.001) higher levels of virus (both peak (Fig 1A and 1B) and total shedding (Fig 1C and 1D) and shed virus for significantly (*P*<0.001) longer periods (Fig 1E and 1F) compared with vaccinated cattle. When considering only carrier animals a similar difference between unvaccinated and vaccinated cattle was identified in all three measures of shedding. However, there was no significant (*P*>0.4) difference in peak shedding, total shedding or duration of shedding for carriers compared with non-carriers within the same group.

On day 21 post challenge only one out of 39 carriers was positive for viral RNA in nasal fluid, but all other samples were negative (i.e. no positives in saliva on day 21 or in nasal fluid and saliva on day 28 post challenge) (Fig 1E and 1F). These results correspond to a proportion of carriers shedding on day 21 of 2.6% (95% confidence interval (CI): 0.1–13.5%) and proportion of carriers shedding in other fluids or at other time-points of 0% (95% CI: 0–9.0%). This difference in proportions is not significant (Fisher exact test: *P* = 1).

### Transmission rate between carrier and sentinel cattle

Based on the outcome of the challenge study (no transmission to two naïve sentinels housed with 9 carriers for 75 days and no transmission to two naïve sentinels housed with 3 carriers for 63 days), the maximum likelihood estimate for the force of infection (λ) is zero, with an upper 95% confidence limit of 0.0022 day^-1^.

### Complete genome sequence analysis

The challenge virus and the isolates from the carrier animals were 8183 nucleotide in length. The poly(C) and poly(A) tracts of FMDV is highly variable and were not sequenced. Ten nucleotides of C were substituted in place of the poly(C) tract during the sequence analysis. The nucleotide identity between the challenge and carrier viruses ranged between 98.90 and 99.77% and the amino acid identity ranged between 99.18% and 99.96% (Table 2).

**Table 2:** Estimates of evolutionary divergence between sequences.

	Challenge virus	UV9-49dpc	UV9-77dpc	UV9-84dpc	UV9-98dpc	UV13-49dpc	UV17-91dpc
**Challenge virus**	***	99.87	99.79	99.74	99.7	99.66	99.53
**UV9-49dpc**	99.61	***	99.91	99.79	99.9	99.53	99.48
**UV9-77dpc**	99.42	99.74	***	99.87	100	99.53	99.4
**UV9-84dpc**	99.38	99.68	99.77	***	99.8	99.48	99.44
**UV9-98dpc**	99.43	99.66	99.69	99.56	***	99.48	99.35
**UV13-49dpc**	99.57	99.17	99.06	99	99.1	***	99.18
**UV17-91dpc**	99.42	99.15	98.99	98.95	98.9	99.04	***

The number of nucleotide and amino acid substitutions was estimated using the Maximum Composite Likelihood and the Poisson correction models as incorporated in MEGA6. The nucleotide and amino acid sequence identities shared among the challenge virus and the viruses/genome obtained from carrier animals are shown. Numbers above the starred diagonal in the Table 2 indicate percent amino acid sequence identity and numbers below the starred diagonal indicate percent nucleotide sequence identity.

**Fig 2:**
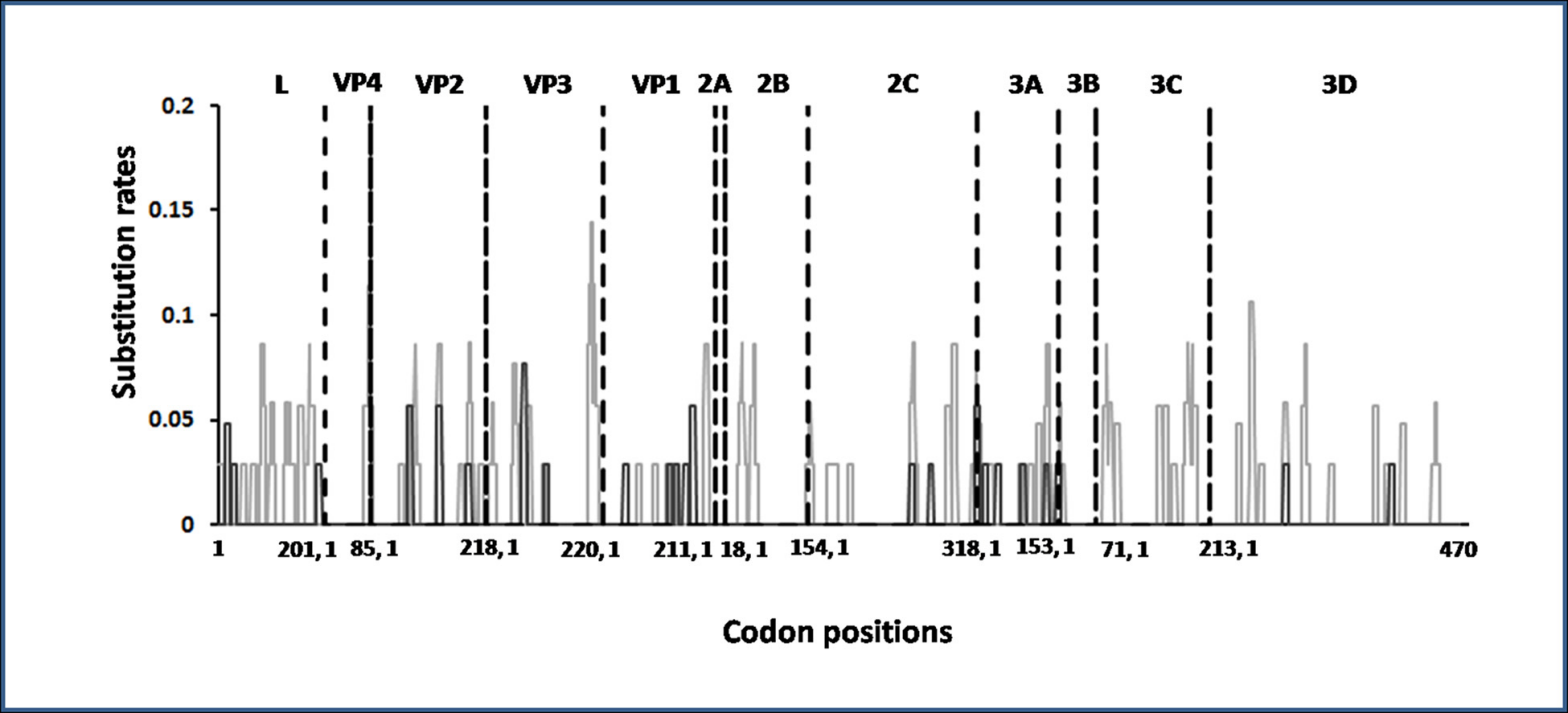
Synonymous (grey) and non-synonymous (black) changes in the ORF of the genomes of the FMD viruses obtained from carrier animals. The black dotted line indicates gene junctions.

The synonymous and non-synonymous changes across the coding region of the different genes of FMDV genome are shown in Fig 2. There was no evidence of positive selection occurring across the genome. Though a total of 24 amino acid substitutions were observed in the protein coding region (ORF) of the carrier isolates, none of them was found in all the isolates (Table 3).

**Table 3:** Amino acid changes in the open reading frames (ORF) of the carrier virus and their relative positions in the viral protein.

Position in ORF	Protein	Position	Change
22	L	22	L-S (2/6)
34	L	34	H-Y (1/6)
194	L	194	A-T (1/6)
365	VP2	79	**Y-H (4/6)**
420	VP2	134	**K-E (3/6)**
475	VP2	189	V-A (1/6)
579	VP3	75	**A-T (5/6)**
580	VP3	76	Q-R (1/6)
620	VP3	116	D-N (1/6)
769	VP1	45	K-E (1/6)
853	VP1	129	V-A (1/6)
865	VP1	141	V-A (1/6)
882	VP1	158	T-M (1/6)
896	VP1	172	**R-Q (3/6)**
1307	2C	200	K-R (1/6)
1343	2C	236	K-R (1/6)
1430	3A	5	**S-T (4/6)**
1445	3A	20	E-D (1/6)
1469	3A	44	Q-H (1/6)
1517	3A	92	E-G (1/6)
1560	3A	135	G-C (1/6)
1582	3B	4	T-I (1/6)
2010	3D	148	K-R (1/6)
2208	3D	346	Y-H (1/6)

The figures in parenthesis in the last column indicate the no. of isolates showing the change out of a total of 6 viruses isolated from the carrier animals in this study. The changes occurring in ≥ 3 viruses are shown in bold.

**Fig 3:**
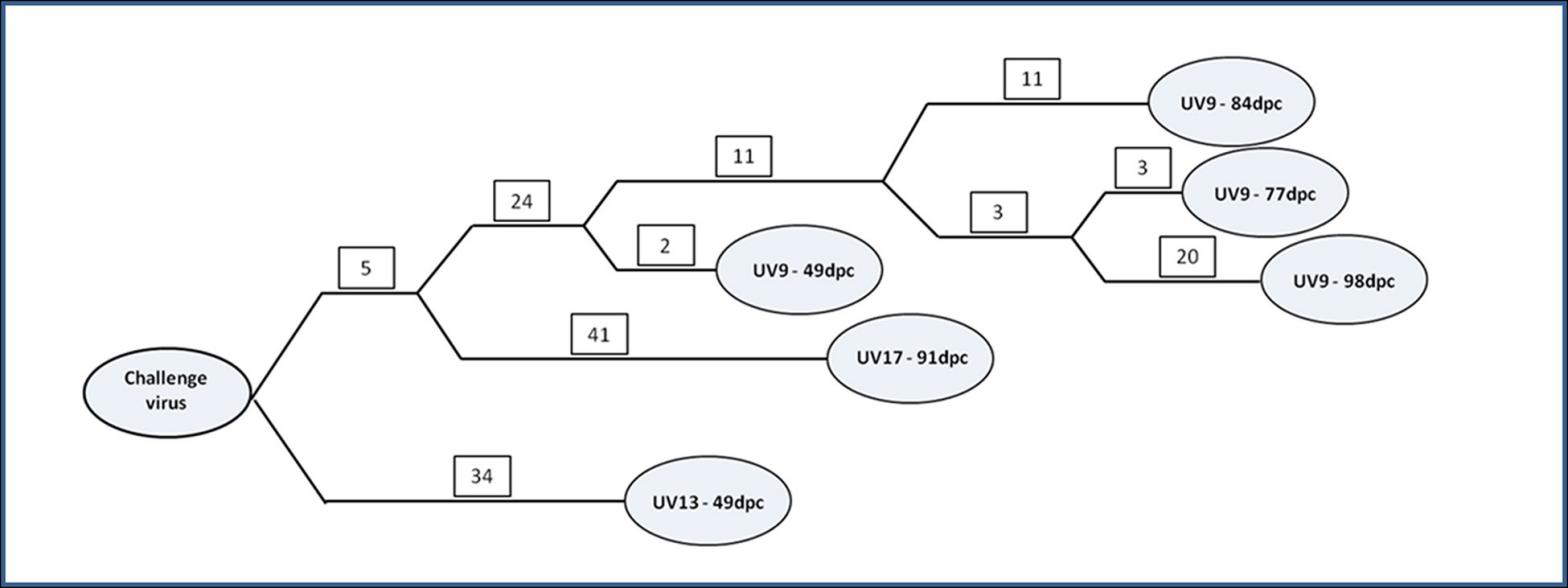
Statistical parsimony analysis of complete genome sequences of challenge virus and viruses/genomes isolated from carrier cattle. The analysis was performed in TCS v1.21. The numbers on the branches represent the number of nucleotide changes (putative ancestral states). The branch lengths are not proportional to time or the number of putative ancestors.

The statistical parsimony analysis revealed that all the carrier isolates originated from the challenge virus. The viruses isolated from UV9 showed 31 nucleotide changes at 49 dpc, 46 nucleotide changes at 77 dpc, 51 nucleotide changes at 84 dpc and 63 nucleotide changes at 98 dpc. The virus isolated from UV13 at 49 dpc showed 34 nucleotide changes and the virus isolated from UV17 at 91 dpc showed 46 nucleotide changes (Fig 3). The parsimony analysis of the complete genomes also shows that the viruses isolated from different carrier animals clustered separately (Fig 3).

The MCC phylogenetic tree showed that the viruses isolated from different animals clustered separately (data not shown), similar to that of the parsimony analysis (Fig 3). The molecular clock rate estimated using the lognormal relaxed clock model for the viruses obtained from carrier animals was found to be 2.6×10^−2^ substitutions per site per year (95% HPD: 2.00×10^−2^ to 3.31×10^−2^). The random local clock model also estimated 2.618×10^−2^ substitutions per site per year (95% HPD: 1.911×10^−2^ to 3.29×10^−2^).

**Fig 4:**
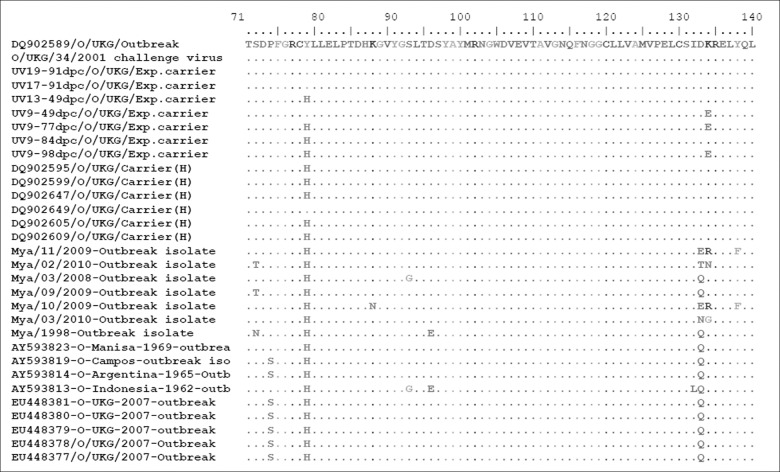
Alignment of VP2 sequences of the FMDV obtained from experimental carrier animals and from animals in acute infection. The substitution of Y to H at VP2_79_ is seen in isolates from both the groups. The sequences marked with (H) are from a previous study by Horsington et al., 2007.

Of the carrier viruses analysed in the present study four (out of six complete genome and one capsid sequence) had the amino acid substitution (Y to H) at the position 79 of the VP2 gene (Fig 4). All 16 viruses isolated from acutely-infected animals in field outbreaks also had the Y to H substitution (Fig 4).

## Discussion

The possibility of transmission of FMDV by recovered, but persistently infected carriers is an important issue for the control of FMDV in both endemic countries and in countries that are disease-free but continue to vaccinate against FMDV. In addition, determining the risk of carrier herds or flocks containing persistently infected individuals which are not detected and removed by conventional means is important in establishing the effectiveness of post-outbreak serosurveillance [32]. The aim of this study was to identify the viral determinants of persistence, explore differences in shedding during the acute phase of infection between carriers and non-carriers and to quantify the transmission rate of FMDV infection from carriers to susceptible animals. As transmission from carriers to susceptible animals occurred under controlled conditions, the dynamics of transmission could be studied without the complexities which make such a study in the field difficult to interpret.

Our analysis of the complete genomes of FMDV isolated from persistently infected animals (3 animals and 6 occasions) showed that the carrier viruses are very similar; with an aminoacid identity in the range of 99.18% to 99.96%. Furthermore, there were no consistent changes in amino acid sequences observed between the challenge virus and these carrier viruses. A previous study reported a substitution (Y to H) at position 79 of the VP2 protein of viruses isolated from five (out of six) persistently infected animals, suggesting this was associated with the ability of the virus to persist [33]. By contrast, we found this substitution in only four (out of seven) viruses isolated from carriers and in another 16 viruses isolated from acutely infected animals in the field, suggesting that this is not a unique change for carrier viruses. In conclusion, we have found no evidence that there are viral determinants influencing persistent infection.

The selection pressures acting on the viral genome varies between individual animals. This is evident from the fact that the isolates showed different routes of evolution in different cattle as shown by the parsimony analysis. This suggests that there are host factors influencing persistent infection.

The mean rate of nucleotide changes in the genome of carrier viruses was estimated to be 2.6×10^−2^ substitutions per site per year, which is similar to values reported previously for the VP1-coding region of viruses isolated from carriers [34]. This rate is, however, higher than typically reported for virus isolated during outbreaks (8×10^−3^ to 9×10^−3^) [35, 36]. A higher mutation rate in carrier viruses could be indicative of a higher selection pressure acting on the FMDV genome in carrier animals.

We found a significant difference between the unvaccinated and vaccinated cattle in peak shedding, total shedding or duration of shedding, but this was independent of whether or not animals subsequently became carriers. This suggests that identifying animals at risk of becoming carriers based on viral shedding during the acute phase is unlikely to be feasible.

Although viral RNA was detected in nasal fluid of a carrier animal on 21 day post challenge, live viruses used in genome sequencing were only recovered when the oro-pharynx was sampled using a probang cup. This might have occurred due to the fact that the usage of probang cup disrupts the cells in oropharynx where the viruses persist, but are not normally shed. This highlights the possible risk of virus spread from the carrier animals to naïve population when the cells harbouring carrier viruses at naso-pharynx and dorsal soft palate become damaged. Further danger of spread of virus from these carrier animals may be possible at the slaughter house when the throats of the animals are exposed, with the possibility of carrier virus being released.

The observation that there was no transmission to two naïve sentinels housed with 9 carrier animals for 75 days and no transmission to two naïve sentinels housed with 3 carriers for 63 days shows that the transmission rate of FMDV from carriers to susceptible animals is zero or at least much lower than the transmission rate estimated during an acute infection of cattle with FMDV [37, 38, 39]. Consequently, the risk of new outbreaks from introduction of a carrier is clearly much lower than the risk of a new outbreak from introduction of an animal during primary infection. This is similar to an earlier observation [20].

Although the probability of FMDV transmission from carrier and the possibility of the carrier animal to initiate an outbreak are very low, the presence of live FMDV in these animals warrants a thorough risk assessment before movement of animals from an area that had recently experienced an outbreak of FMD. This risk of introduction of virus into a susceptible population by means of a carrier can be achieved by increasing the interval between the last outbreak and the time of import [20]. Such a population need to be screened by established laboratory methods and epidemiological investigation.

## Supporting Information

S1 Table Foot-and-mouth disease virus genome/ capsid sequenced in this study and their GenBank accession numbers. (DOCX)

S2 Table Primers used for amplifying the complete genome of FMDV. (DOCX)

S3 Table Primers used for sequencing the complete genome of FMDV. (DOCX)

S4 Table qRT-PCR analysis for the presence of FMDV genome in the nasal swabs of both vaccinated and unvaccinated challenged cattle. (XLSX)

S5 Table qRT-PCR analysis for the presence of FMDV genome in the saliva swabs of both vaccinated and unvaccinated challenged cattle. (XLSX)
