# Supplementary material for: Virus Excretion from Foot-And-Mouth Disease Virus Carrier Cattle and Their Potential Role in Causing New Outbreaks
Source: PLoS One. 2015 Jun 25;10(6):e0128815. doi: 10.1371/journal.pone.0128815 (PMC4482020; doi:10.1371/journal.pone.0128815)
Supplement: S1 Table — (DOCX) [file pone.0128815.s001.docx]

**Supplementary Table1: Foot-and-mouth disease virus genome/capsid sequenced in this study and their GenBank accession numbers**

| **Name of the isolate** | **Accession Number** |
| --- | --- |
| O/UKG/ UV9/49dpc | KR265072 |
| O/UKG/ UV9/77dpc | KR265073 |
| O/UKG/UV9/84dpc | KR265074 |
| O/UKG/UV9/98dpc | JX947859 |
| O/UKG/UV13/49dpc | JX947860 |
| O/UKG/UV17/91dpc | JX947858 |
| O/UKG/Challenge Virus | KR265075 |
| O/UKG/UV19/91dpc | KR265076 |
| O/MYA/01/1998 | KR265077 |
| O/MYA/03/2008 | KR265078 |
| O/MYA/09/2009 | KR265079 |
| O/MYA/10/2009 | KR265080 |
| O/MYA/11/2009 | KR265081 |
| O/MYA/02/2010 | KR265082 |
| O/MYA/03/2010 | KR265083 |
