# Supplementary material for: Virus Excretion from Foot-And-Mouth Disease Virus Carrier Cattle and Their Potential Role in Causing New Outbreaks
Source: PLoS One. 2015 Jun 25;10(6):e0128815. doi: 10.1371/journal.pone.0128815 (PMC4482020; doi:10.1371/journal.pone.0128815)
Supplement: S2 Table — (DOCX) [file pone.0128815.s002.docx]

| Segment – 1 | Forward1 | TTG AAA GGG GGC GTT AGG GTC TCA |
| --- | --- | --- |
|  | Reverse1 | GGG TGA AAG GTG GGC TTY GGG T |
| Segment – 2 | Forward2 | CCC AAG TTT TTA CCG CCT TTC CCG |
|  | Reverse2 | GTT GAT AAT GCT TCC AGT GTT GCC TG |
| Segment – 3 | Forward3 | CCA CGC TGG CAT CTT CCT GAA AG |
|  | Reverse3 | GAG CTT GTA CCA GGG TTT GGC CTC A |
| Segment – 4 | Forward4 | GTG TTG GAC CTG ATG CAA ACC CC |
|  | Reverse4 | GTC TCT TGC GAG TCT CGC GGA TC |
| Segment – 5 | Forward5 | TTC AAG CCT CAA CCG CCC CTC |
|  | Reverse5 | GGC GGC CGC TTT TTTTTTTTTTTT |

**Supplementary Table 2. Primers used for amplifying the complete genome of FMDV**
