# Supplementary material for: Virus Excretion from Foot-And-Mouth Disease Virus Carrier Cattle and Their Potential Role in Causing New Outbreaks
Source: PLoS One. 2015 Jun 25;10(6):e0128815. doi: 10.1371/journal.pone.0128815 (PMC4482020; doi:10.1371/journal.pone.0128815)
Supplement: S3 Table — (DOCX) [file pone.0128815.s003.docx]

**Supplementary Table 3: Primers used for sequencing the complete genome of FMDV**

| FOR1 | TTGAAAGGGGGCGTTAGGGTC | REV1 | GAAATAGGAAGCGGGAAAAAGCC |
| --- | --- | --- | --- |
| FOR2 | CGTGACTGGTTAATACTCTTACC | REV2 | GGTCTTCGAAGCCATCACAG |
| FOR3 | CCAAGTTTTTACCGCCTTTCCC | REV3 | CTTGCAACCACGATGTCGTC |
| FOR4 | GCTTGAGGAGGACTTGTACAAAC | REV4 | TCAGGATCCACTCAGCGTTTG |
| FOR5 | AGCACTGTTGCTTCGTAGCGG | REV5 | CACAAAATCTGCCAATCATCATC |
| FOR6 | CACTGGTGACAGGCTAAGGATG | REV6 | ACCACGGCGTTTCCCCTG |
| FOR7 | CACTGTTCTTATCACGAGCAC | REV7 | CCTCCTCAGACATCTTTGTGTTTC |
| FOR8 | GATGCTATCAAACAATTGGAAG | REV8 | GTGTGGTTCGGGATCGATGTG |
| FOR9 | GAGGACTTTTACCCCTGGACG | REV9 | TCCGTCCATGCACACTACAATG |
| FOR10 | CAATTACTACATGCAGCAGTACCAG | REV10 | GAGCATGTCCTGTCCTTTCAC |
| FOR11 | ACCGAGGAGACCACTCTTCT | REV11 | GTCGAGGATGAGCTCAACAG |
| FOR12 | GTTCTTCAAAACCCACTTGTTC | REV12 | GTTTCTGTCTCTCCATCGGACC |
| FOR13 | CAGAACTTTGCTCTATTGACAAG | REV13 | GGTCTCCAGAGGGTTCTTTTCC |
| FOR14 | CAACATCGCCCCTACCAACG | REV14 | CTCAAAGTTTTCCTTCAGGCG |
| FOR15 | GTTCACCAACTTCCTTGATG | REV15 | ACCTTCTCGTGGAGCTCGAC |
| FOR16 | CACCATCAACCTGCACTTCATG | REV16 | TTGGCGCTCACGTCAATGTC |
| FOR17 | GGAGACCACAAATGTACAGG | REV17 | CCGTAGTTGAAACCATTTGGG |
| FOR18 | GTTGAGAACTACGGTGGTGAG | REV18 | AGCACGTTCGCAAGGAAACTC |
| FOR19 | CTAGAAGTGGCAGTGAAACAC | REV19 | GCTGCTTTTCAAGGATGCC |
| FOR20 | GTGAGAGGTGACCTGCAAG | REV20 | CTCTCAAGGTCTTCGGGTGTG |
| FOR21 | GCTCAAGTTGGCAGGAGACG | REV21 | GTCCTTTGACCGTGCTGCTAC |
| FOR22 | CCACTGGAGTGAAGGCTATCAG | REV22 | GACACCAACCGGTTAAAGTCG |
| FOR23 | GAAGATCTCCGACTCGCTCTCC | REV23 | CCTCTTCATGCGGTAAAGCAG |
| FOR24 | CCATTCTCAAGAACGGCGAGTG | REV24 | CCGTGTAAGGCAGTGCAAGC |
| FOR25 | GTGTTTGAAGAGCGGGAACATC | REV25 | GGGTTTGCATCAGGTCCAAC |
| FOR26 | ACCTGACCCTGACCACTTCGAC | REV26 | GCTAGCTAGAACGACCAGTGCG |
| FOR27 | CAACCTGTACTCGGGCTTCAC | REV27 | TGTCCCACTCCGCATGAATG |
| FOR28 | ACGACTGTGCCCTTCTCAACG | REV28 | CTGTCTGAGTCCGTCTTTGTGG |
| FOR29 | CTTTGAGGGGATGGTGCATGAC | REV29 | ACCACCGTAACCGTCGCTAC |
| FOR30 | CAAGAGACAGCAGATGGTGGATG | REV30 | CCAACAAAGGGCACAGTGATG |
| FOR31 | GAAGAACAACCACAAGCTGAAGG | REV31 | CAACCGTTTCTCATGTAAGCATAAG |
| FOR32 | GAAGAAACCTGTCGCTTTGAAAG | REV32 | GCTGTTGCGTACCCGTAAGTG |
| FOR33 | GACAAGATCATGTTGGACGG | REV33 | CCAGTCATTGTTCTGAGTGTTGG |
| FOR34 | ACGCTGATGTTGGGAGACTG | REV34 | CTTTCCATTCTCCGTTGAGC |
| FOR35 | CTTTCATCGTCGGCACTCAC | REV35 | GTCCCGTCTACCATGCACAC |
| FOR36 | GGTGTGTTTAACCCCGAATTTGG | REV36 | GGTTCATCAACGTACCTAAACAAC |
| FOR37 | TGCATAGCGTGCTGGGTACG | REV37 | GGTCACCTATTCAGGCGTAGAAG |
| FOR38 | CTTCCTGAAGGACGAGATTCGC | REV38 | GTTGCACACATGGTGGGTC |
| FOR39 | GATTTGGCACGCATTTTGCTC | REV39 | CCCCTCTAGACCTGGAAAGACC |
| FOR40 | TTGTTCCGCAACAAGCATCATC | REV40 | GGGAAAGGCGGTATCATCTTG |
| FOR41 | CCAGCTGACAAAAGCGACAAAG | REV41 | GGGTGAAAGGTGGGCTTCG |
| FOR42 | TCTGGACCTGACGAGTACCG | REV42 | GTTCGTCCGCACAGTCCAG |
